# Magnetic biocatalysts and their uses to obtain biodiesel and biosurfactants

**DOI:** 10.3389/fchem.2014.00072

**Published:** 2014-08-26

**Authors:** Carmen López, Álvaro Cruz-Izquierdo, Enrique A. Picó, Teresa García-Bárcena, Noelia Villarroel, María J. Llama, Juan L. Serra

**Affiliations:** ^1^Enzyme and Cell Technology Group, Department of Biochemistry and Molecular Biology, Faculty of Science and Technology, University of the Basque Country (UPV/EHU)Bilbao, Spain; ^2^IKERBASQUE, Basque Foundation for ScienceBilbao, Spain

**Keywords:** magnetic nanoparticles (MNPs), magnetic cross-linked enzyme aggregates (mCLEAs), biodiesel, biosurfactants, sucrose monopalmitate

## Abstract

Nanobiocatalysis, as the synergistic combination of nanotechnology and biocatalysis, is rapidly emerging as a new frontier of biotechnology. The use of immobilized enzymes in industrial applications often presents advantages over their soluble counterparts, mainly in view of stability, reusability and simpler operational processing. Because of their singular properties, such as biocompatibility, large and modifiable surface and easy recovery, iron oxide magnetic nanoparticles (MNPs) are attractive super-paramagnetic materials that serve as a support for enzyme immobilization and facilitate separations by applying an external magnetic field. Cross-linked enzyme aggregates (CLEAs) have several benefits in the context of industrial applications since they can be cheaply and easily prepared from unpurified enzyme extracts and show improved storage and operational stability against denaturation by heat and organic solvents. In this work, by using the aforementioned advantages of MNPs of magnetite and CLEAs, we prepared two robust magnetically-separable types of nanobiocatalysts by binding either soluble enzyme onto the surface of MNPs functionalized with amino groups or by cross-linking aggregates of enzyme among them and to MNPs to obtain magnetic CLEAs. For this purpose the lipase B of *Candida antarctica* (CALB) was used. The hydrolytic and biosynthetic activities of the resulting magnetic nanobiocatalysts were assessed in aqueous and organic media. Thus, the hydrolysis of triglycerides and the transesterification reactions to synthesize biodiesel and biosurfactants were studied using magnetic CLEAs of CALB. The efficiency and easy performance of this magnetic biocatalysis validates this proof of concept and sets the basis for the application of magnetic CLEAs at industrial scale.

## Introduction

The use of nanobiocatalysts, with the combination of nanotechnology and biocatalysis, is considered as an exciting and rapidly emerging area. Thus, nanobiocatalysis, as a new frontier of biotechnology, is a new innovative sub-field of biocatalysis which explores more advanced materials as enzyme carriers as well as provides robust nanostructured materials with properties tailored to their applications as enzyme scaffolds (Xin et al., 2010).

One of the great challenges that the industries face nowadays is the transition to greener and more sustainable manufacturing processes that minimize, or preferably avoid, the generation of waste and the use of toxic and/or hazardous materials. Biocatalysis has many benefits to offer in this respect, since enzymatic processes generate less waste than conventional synthetic routes, are more energy efficient, and provide products in higher purity (Sheldon, 2011).

Enzymes are versatile nanoscale biocatalysts which can be used in many areas of application, including industrial biocatalysis and bioremediation. However, the use of enzymes in soluble form is often hampered by their price, instability and the difficulty in its recovery and reutilization. These drawbacks can generally be overcome by immobilizing the enzyme to solid supports, since the immobilized biocatalyst shows improved storage and operational stability (e.g., toward denaturation by heat or organic solvents or by autolysis) and it can be easily separated from the products in the reaction mixture and reused. Moreover, reutilization of enzyme in consecutive catalytic cycles significantly decreases the costs of the biocatalyst which otherwise would not have been economically viable using the free enzyme (Sheldon, 2011; Sheldon and van Pelt, 2013).

Recent advances in nanotechnology provide a range of more diverse nanomaterials and the approach to immobilize enzymes on these nanosupports has grown in popularity in recent years. At the present time, the use of iron oxide magnetic nanoparticles (MNPs) as enzyme immobilization carriers, has drawn great attention because of their unique properties, such as controllable particle size, large surface area, modifiable surface, and easy recovery by applying a magnetic field which allows its reuse for successive catalytic cycles (Johnson et al., 2011; Liese and Hilterhaus, 2013; Verma et al., 2013; Kopp et al., 2014).

Enzyme immobilization on magnetic supports was first reported by Matsunaga and Kamiya (1987) who used magnetic particles isolated from magnetotactic bacteria. Later Dyal et al. (2003) reported magnetic (maghemite) nanoparticles for the immobilization of *Candida rugosa* lipase. Further progress in the use of magnetic materials for enzyme immobilization has been dependent on developments in MNP synthesis/handling and control over magnetic properties (Yiu and Keane, 2012).

Magnetic nanomaterials greatly facilitate separation, allowing the use of a magnet to quickly and efficiently remove the immobilized enzyme from the product (Safarik and Safarikova, 2009; Ren et al., 2011). This allows greater reusability and preservation of stability of the attached enzyme as compared to conventional matrices, where centrifugation/filtration is the only option to separate the enzyme from the product. Such operations might lead to enzyme leaching/instability due to mechanical shear while mixing the pellet with the appropriate buffer to begin a new reaction (Yiu and Keane, 2012). The low process costs of magnetic nanocarriers have therefore shown them to be an interesting and economic option (Verma et al., 2013).

In the past couple of decades, cross-linked enzyme aggregates (CLEAs) have emerged as a novel and versatile carrier-free immobilization technique (Cao et al., 2000; Sheldon, 2011). Moreover, the use of CLEAs presents several advantages compared to the free enzyme, since they are more stable with temperature and show good reusability, retaining a high percentage of their initial activity after several cycles. The preparation of CLEAs involves the precipitation of the enzyme (that does not need to be pure) and subsequent chemical cross-linking of the resulting protein aggregates with glutaraldehyde. This bi-functional reagent is generally the cross-linker of choice as it is inexpensive and readily available in commercial quantities (Sheldon and van Pelt, 2013).

Despite the advantages of CLEAs, the number of enzymes immobilized by this technology is limited, mainly due to the low Lys residue contents in the external surface of some enzymes (Sheldon, 2007), and the increased size (clumping) of CLEAs clusters due to separation of CLEAs from reaction mixture by centrifugation or filtration (Montoro-García et al., 2010; Wang et al., 2011). The latter limitation can be overcome if the CLEAs are magnetically-separable and their recovery can be easily achieved using a magnet instead of using centrifugation or filtration methods which inevitably lead to clumping of CLEAs. mCLEAs of α-amylase from *Bacillus* sp. (Talekar et al., 2012) and lipase from *Aspergillus niger* (Tudorache et al., 2013) were successfully prepared and used to hydrolyze starch and to obtain glycerol carbonate, respectively.

Biodiesel is as a mixture of fatty acid alkyl esters (FAAEs) which can be produced by transesterification of oils or by esterification of free fatty acids (FFAs) catalyzed either chemically or enzymatically using a lipase. Chemically-catalyzed production of biodiesel is industrially acceptable for its high conversion and reaction rates. However, downstream processing costs, environmental issues associated with biodiesel production and byproducts recovery have led to the search for alternative more eco-friendly production methods (Bisen et al., 2010). Thus, lipase-mediated biodiesel production presents more advantages over the chemical method since it is eco-friendly, chemically selective and requires lower temperatures (Verma et al., 2013).

Sugar fatty acid esters (SFAEs), synthesized from renewable resources, have broad applications in detergent, food and cosmetic industries (van Kempen et al., 2013). Moreover, these biodegradable biosurfactants present antitumor, antimicrobial and insecticidal properties. SFAEs can be synthesized by chemical methods, although these reactions must be performed at high temperature and pressure in alkaline media and result in poor selectivity and colored side-products (Huang et al., 2010; Gumel et al., 2011; van den Broeck and Boeriu, 2013). SFAEs were also enzymatically synthesized using immobilized lipase and obtaining high production yields (Ferrer et al., 2002), with recovery of the granulated enzyme by decantation.

In this work, by using the aforementioned advantages of MNPs of magnetite and those of CLEAs, we prepared two robust magnetically-separable types of nanobiocatalysts by binding either the soluble lipase B of *Candida antarctica* (CALB) onto the surface of MNPs functionalized with –NH_2_ groups (MNP-CALB) or by cross-linking with glutaraldehyde aggregates of enzymes among themselves and to MNPs to obtain magnetic CLEAs (mCLEA-CALB). Both biocatalysts were used to obtain: (i) fatty acid ethyl and propyl esters (biodiesel) by esterification of FFAs and transesterification of non-edible vegetable oils; (ii) sucrose monopalmitate (biosurfactant) from sucrose and vinyl, ethyl and methyl palmitate. The rapid magnetic recovery of the biocatalysts, their stability and the simple reaction media are exploited to establish an enzymatic process which could be easily transferable to industrial scale.

## Materials and methods

### Materials

3-Aminopropyltriethoxysilane (APTS), NaBH_4_, FeCl_2_, FeCl_3_, dimethylsulfoxide (DMSO), 2-methyl-2-propanol (2M2P), *p*-nitrophenyl acetate (*p*NPA), 4 Å molecular sieves and Triton X-100 were purchased form Sigma-Aldrich (St. Luis, MO, USA). Coomassie Blue (PhastGel™ Blue R) was obtained from GE Healthcare (Uppsala, Sweden). Vinyl, methyl and ethyl palmitate and FFAs were purchased from TCI Chemicals (Portland, OR, USA). Non-edible vegetable oils (unrefined soybean, jatropha and cameline) were obtained from Bunge Ibérica, S.A. (Zierbena, Spain), Jatropha Hispania, S.L. (Toledo, Spain), and Camelina Company (Madrid, Spain), respectively. Olive oil used as a control was purchased from Carbonell (Madrid, Spain). All other chemicals were supplied by Merck (Darmstadt, Germany).

### Enzyme

Lipozyme® CALB L, lipase B of *C. antarctica* (CALB, EC 3.1.1.3, 19.1 *U*/mg protein; 7.50 mg protein/ml) was kindly provided by Novozymes (Bagsvaerd, Denmark).

### Synthesis and functionalization of magnetic nanoparticles (MNPs)

MNPs of magnetite (Fe_3_O_4_) were synthesized by coprecipitation of iron salts in alkaline medium following the method described by Cruz-Izquierdo et al. (2012). Briefly, an aqueous solution containing 0.36 M FeCl_2_ and 0.72 M FeCl_3_ was pumped to 1 M NH_4_OH solution under continuous mechanic stirring at room temperature. The obtained black precipitate was separated from the liquid phase using a magnetic field, and then magnetically washed with water and PBS buffer (100 mM sodium phosphate, 150 mM NaCl, pH 7.4). In order to functionalize the resulting MNPs with –NH_2_ groups, MNPs were incubated with APTS, washed with PBS and maintained at 4°C until use.

### Preparation of immobilized enzymes on MNPs

MNP–NH_2_ (20 mg dry weight) was functionalized with aldehyde groups by incubation with 250 mM glutaraldehyde for 4 h in a final volume of 10 ml (phosphate buffer solution 100 mM, pH 7.4). The protein (50 μ g/mg MNPs) was added to the mixture, which was maintained overnight at 4°C. After the immobilization, the MNP-enzyme was washed with 2 M NaCl and 1% Triton X-100 (v/v) in order to remove ionic and hydrophobic interactions, respectively. Finally, the enzyme was washed with buffer and maintained at 4°C.

### Preparation of magnetic CLEAs

mCLEAs of CALB (mCLEA-CALB) were prepared following the methodology proposed by Cruz-Izquierdo et al. (2012). MNP–NH_2_ was incubated with the corresponding enzyme to obtain 25–100 μg CALB/mg MNP–NH_2_ in the presence of 100 mmol ammonium sulfate/mg protein. After 10 min, a solution of glutaraldehyde was added to reach a final concentration of 12.5 mM and the suspension was maintained for 5 h in agitation. mCLEAs were then washed with 2 M NaCl, 1% (v/v) Triton X-100 and 200 mM bicarbonate buffer, pH 9.0.

### Characterization of MNPs and immobilized enzymes

Elemental analysis was performed using a Euro EA Elemental Analyzer CHNS (EuroVector, Milan, Italy) with quantification limit of 0.1%. The particle size and morphology of MNPs was determined by transmission electron microscopy (TEM, JEOL 1010, Peabody, MA, USA). The magnetic characteristics were measured using different devices: for the calculation of magnetic saturation (*M*_*s*_) 2 K hysteresis loops were performed using a vibrating sample magnetometer (VSM) in a superconducting magnet (14 T) cooled by a closed circuit of He (CFMS, Cryogenic Ltd., London, UK). To calculate coercivity (*H*_*c*_) and remanence (*M*_*r*_) an electromagnet was used at room temperature and moderate fields (0.9 T). The size distribution of magnetic particles was calculated from the magnetization data according to Langevin's equations adjusted for non-interacting superparamagnetic model. MNPs dry weight and concentration were analyzed using a vacuum concentrator (Savant SpeedVac concentrator, Thermo Scientific, Waltham, MA, USA).

### Enzymatic activity measurements

#### Esterase activity of CALB

The activity of soluble and immobilized CALB was assayed with *p*NPA as substrate according to Gao et al. (2004) with minor modifications. Specifically, soluble or immobilized lipase (1–2 μ g protein) was added to a reaction mixture which contained 10 μl of *p*NPA (100 mM in DMSO) in 980 μl of PBS. The reaction mixture was maintained for 15 min at room temperature with rotational mixing at 30 rpm (Intelli-Mixer RM-2, Elmi Ltd., Riga, Latvia). Samples were withdrawn every 5 min (in the case of immobilized lipase the magnetic biocatalysts were separated from the liquid phase using a magnet) and the appearance of *p*-nitrophenol (*p*NP) was measured spectrophotometrically (Beckman Coulter DU 800, Brea, CA, USA) at 405 nm (ε_*pNP*_ = 9.43 mM^−1^ · cm^−1^). One unit (*U*) of esterase activity was defined as the amount of enzyme that catalyzes the appearance of 1 μmol *p*NP per min under the assay conditions.

#### Hydrolytic activity of CALB

The hydrolytic activity of CALB was analyzed by the hydrolysis of tributyrin, and the release of butyric acid was volumetrically measured using a pH-stat (Metrohm 842 pH-Stat Autotitrator, Herisau, Switzerland) with 10 mM NaOH as alkaline solution. CALB (0.01 mg of free enzyme, 1 mg of MNP-CALB or 0.25 mg of mCLEA-CALB) was incubated in 9 ml of 5 mM phosphate buffer, pH 7.3, at 30°C. Tributyrin was then added (0.5 ml) and the increase of pH was registered. One unit (*U*) of activity was defined as the amount of enzyme that catalyzes the release of 1 μmol butyric acid per min under the assay conditions.

#### Transesterification activity of CALB

Olive oil (0.2 g) and 2-propanol (1:6 molar ratio oil:alcohol) were incubated with 1% (w/w of oil) of magnetic catalyst. The reaction was maintained at 40°C and 30 rpm with rotational mixing. The initial reaction rate for biodiesel production was assessed by withdrawing aliquots (5 μl) of the liquid phase at defined intervals which were analyzed by high-performance liquid chromatography (HPLC) as indicated below. Transesterification activity was defined as mg of oil transformed to biodiesel (fatty acid propyl esters, FAPEs) per mg of MNP-NH_2_ and time (h) considering the initial reaction rate of transesterification.

### Synthesis of bioproducts

#### Synthesis of biodiesel

Biodiesel (Fatty Acid Ethyl Esters, FAEEs) was obtained by esterification of FFAs. For that, a mixture of FFAs which simulates the composition of FFAs coming from microalgae (*Scenedesmus* sp.) was applied: 4.1% stearic acid, 23.6% palmitic acid, 52.1% oleic acid, 12.4% linoleic acid, and 7.8% linolenic acid. Ethanol (10:1 alcohol:FFA, mol:mol) was added to FFAs mixture (500 μmol) and 2 mg mCLEA-CALB. FAEEs were also obtained by transesterification of a mixture (0.2 g) of vegetable oils, composed of 80% palm oil, 10% soybean oil and 10% olive oil, with ethanol as alkyl donor in molar ratio of 30:1 (alcohol:oil) and 1% (w/w of oil) of magnetic biocatalyst. FAPEs were produced by transesterification of vegetable oil (0.2 g) using 2-propanol as alkyl donor (6:1 molar ratio alcohol:oil) and 1% (w/w of oil) of magnetic catalyst. In all the cases, the reactions were carried out in a solvent-free medium at 25°C with rotational mixing (Intelli-Mixer RM-2, Elmi Ltd., Riga, Latvia). The reactions were followed by withdrawing aliquots (10 μl) of the liquid phase. Semi-quantitative analyses of samples were performed by thin layer chromatography (TLC) and the quantitative analysis using control FFAs and olive oil was performed by HPLC, as described above.

#### Synthesis of biosurfactant

Sucrose 6′-monopalmitate (SMP) was synthesized by transesterification of sucrose and vinyl, methyl or ethyl palmitate catalyzed by mCLEA-CALB. Sucrose (100 mg/ml) was added to DMSO and stirred for 10 min at 70°C, or to 2M2P and stirred for 24 h at 70°C, in order to obtain a homogeneous mixture. Molecular sieves (4 Å) were added to solid sucrose, DMSO and 2M2P stocks in order to absorb their water content. Vinyl, methyl or ethyl palmitate was then added in a molar sucrose:alkyl palmitate ratio of 1:1, 1:2, or 1:3. mCLEA-CALB were washed with the corresponding solvent and used as catalysts in a concentration of 4 mg/ml. The reactions (5 ml) were incubated at 60°C and stirred using magnetic agitation (Carousel 12 Plus, Radleys, UK). Samples (100 μl) were withdrawn and the liquid phase was separated from the solid biocatalyst using a magnetic field. When using DMSO, the same volume of ethanol was added to the aliquots in order to overcome the immiscibility between palmitate esters and DMSO. Semi-quantitative and quantitative analyses of samples were performed by TLC and HPLC-MS, respectively, as described above.

### Analytical methods

#### HPLC analysis of biodiesel

Five microliter of samples withdrawn from the reaction mixture were diluted in 250 μl of *n*-hexane and analyzed by HPLC (Waters™ Corporation, Milford, MA, USA**)** using a diode array detector according to Holčapek et al. (1999). The C_18_ column (5 μm, 4.6 × 250 mm, Tracer Lichrosorb RP18) was eluted at a flow rate of 1 ml/min using a mixture of acetonitrile:H_2_O (1:1 v/v) (phase A) and 100% pure methanol (phase B) as mobile eluting phase and a gradient from 75 to 100% phase B in 15 min. The injection volume was 10 μl and peaks were detected at 205 nm.

#### HPLC-MS analysis of biosurfactants

Analysis of SMP was carried out by HPLC (Alliance e2695, Waters™ Corporation, Milford, MA, USA) coupled with a triple quadrupole masses spectrometer (QqQ) (Quattro micro Api, Waters™ Corporation, Milford, MA, USA). The column (2.6 μm, 2.10 × 50 mm, Kinetex C18, Phenomenex) was eluted at 30°C and 0.2 ml/min flow-rate. 0.1% (v/v) formic acid in H_2_O (phase A) and 0.1% (v/v) formic acid in methanol (phase B) were employed as mobile phase, with a gradient from 50 to 100% phase B in 9 min. The injection volume was 10 μl. For the ionization of the SMP, a positive electrospray was used with 3200 V capillary voltage at 120°C. Nitrogen was used as desolvation gas at 300°C and 450 l/h flow-rate. SMP was monitorized using MRM (multiple reaction monitoring) with a lineal calibration (10–500 ng/ml) following the next transition: 603.2 to 441.3 nm (for quantification) and 603.2 to 203.1 nm.

#### Thin layer chromatography (TLC) analysis

A semi-quantitative analysis of biodiesel and biosurfactant was also assessed using TLC. Silica gel 60 coated plates (Merck, Darmstadt, Germany) were activated for 30 min at 100°C and 0.5 μ l samples were applied. For biodiesel analysis, the ternary mixture *n*-hexane:ethyl acetate:acetic acid (90:10:1, v/v/v) was used as eluting phase (Samakawa et al., 2000). After chromatography development (about 40 min), plates were air dried at room temperature, and then immersed for 1 min with gentle orbital shaking in a 0.02% (w/v) solution of Coomassie Blue R-350 (Nakamura and Handa, 1984), prepared in acetic acid:methanol:H_2_O (1:3:6, v/v/v) as indicated by the manufacturer. Finally, the plates were air-dried at room temperature. Spots corresponding to substrates and products of the transesterification reaction were identified by using appropriate reference external standards run in parallel. For biosurfactant analysis, the eluting phase was composed of a mixture of toluene:ethyl acetate:ethanol (2:1:1, v/v/v). Spots corresponding to sucrose and SMP were detected by spraying the plates with a solution of urea (1 g urea, 4.05 ml phosphoric acid and 48 ml water-saturated 1-butanol) and heating them at 100°C for 15 min.

## Results and discussion

### Characterization of MNPs and immobilized enzymes

**Figure 1:**
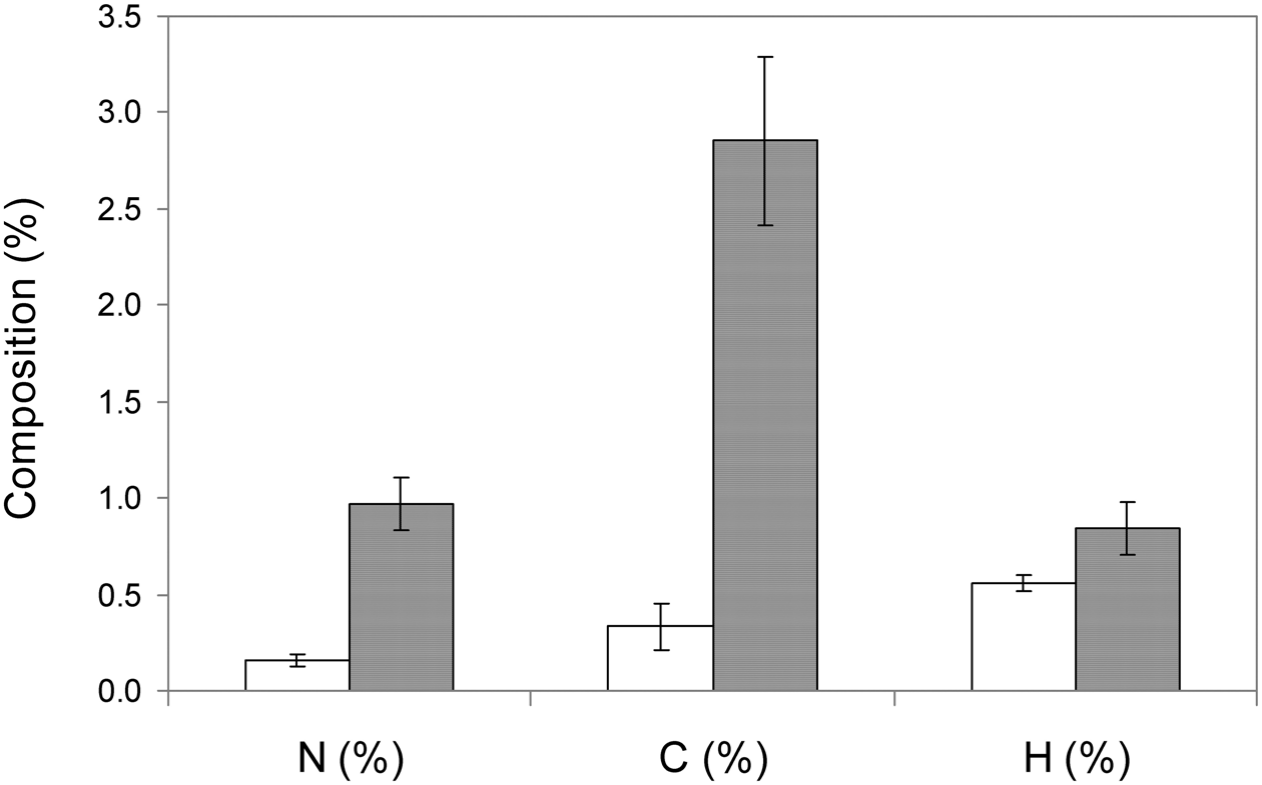
**Elemental analysis of naked MNPs (white bars) and MNP–NH_2_ (gray bars)**.

MNPs were synthesized in our laboratory and analyzed by X-ray diffractography, which confirmed the presence of magnetite (data not shown). The increase of N content in MNPs with –NH_2_ groups was confirmed by comparing the content of N, C, and H in naked MNPs and MNP-NH_2_ using elemental analysis (see Figure 1). Percentage of N was negligible in non-functionalized nanoparticles, and increased up to 1% in particles whose surfaces were coated with groups. The increase was consistent with the presence of amine groups in the MNP–NH_2_. The increment of C content in functionalized nanoparticles was also noticeable, and could correspond to the C content of the functionalizing agent (APTS) used.

**Figure 2:**
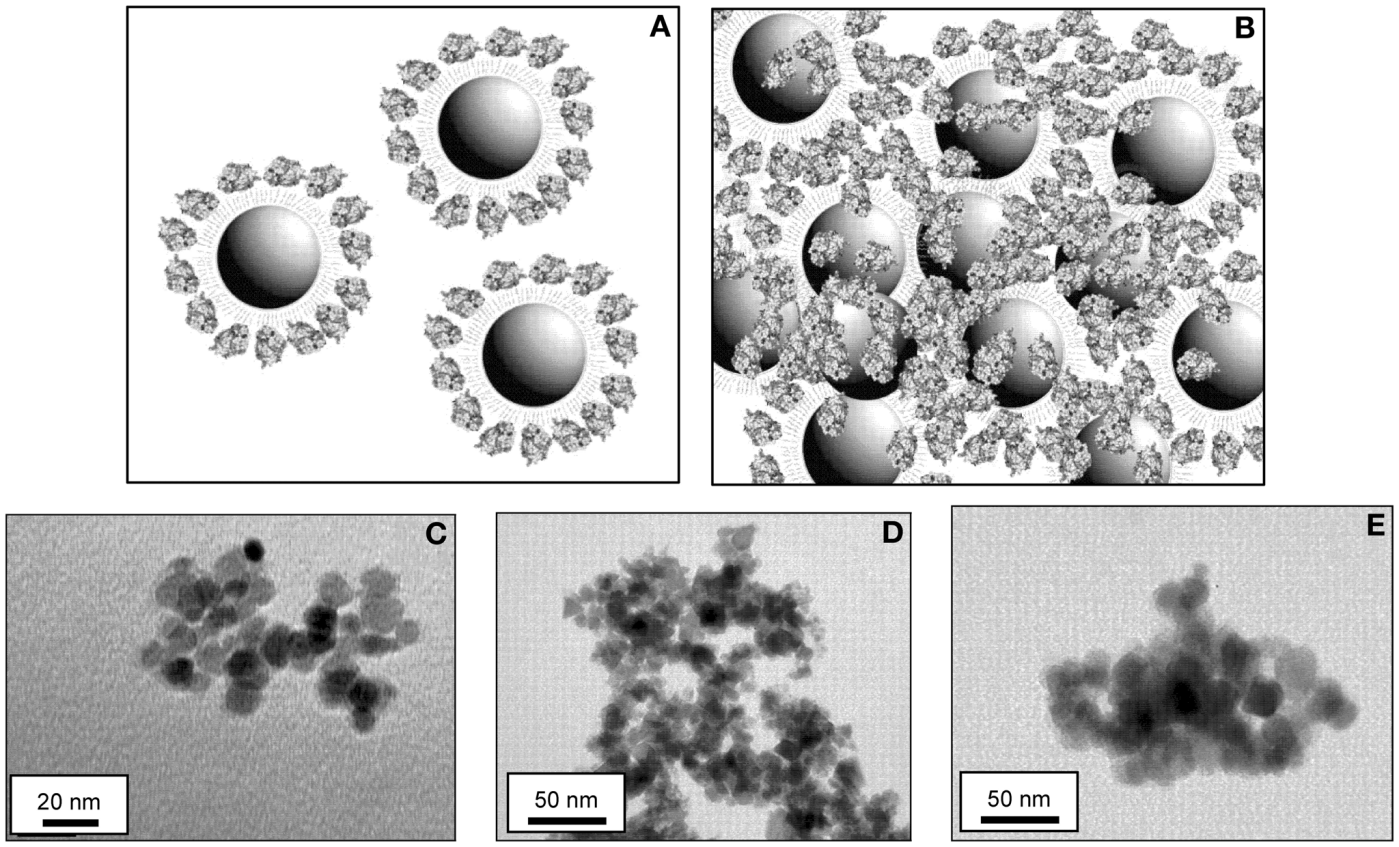
**Scheme of a scale representation of the magnetic biocatalysts obtained with CALB and TEM micrographs of the magnetic biocatalysts of CALB used in this work**. Artwork is a hypothetical model of **(A)** MNP-CALB and **(B)** mCLEA-CALB complexes. Both models simulate a cross-section of a three-dimensional structure. The MNPs are 10 nm in diameter, the APTS/glutaraldehyde bridge provides a spacer arm of 2.5 nm (Dauphas et al., 2009) and the size of CALB is 3× 4 × 5 nm (Uppenberg et al., 1994). TEM images of naked MNPs **(C)**, MNP-CALB **(D)** and mCLEA-CALB **(E)**. Bars represent 20 nm in **C**, and 50 nm in **D,E**.

The functionalized MNP-NH_2_ were then employed for the immobilization of CALB by two different procedures: (i) covalent immobilization by cross-linking of the protein on the surface of MNP-NH_2_ (MNP-CALB, see Figure 2A); (ii) covalent immobilization of cross-linked enzyme aggregates (mCLEA-CALB, see Figure 2B). In both cases glutaraldehyde was used as a bifunctional cross-linking reagent. Both types of immobilized enzymes of CALB were selected as model biocatalysts for characterization purposes.

The shape and size of MNPs and resulting immobilized enzymes were analyzed by TEM. Naked MNPs appeared as spherical particles with a uniform and defined size (see Figure 2C). Because the protein is not as opaque to electrons as MNPs, the presence of CALB bound directly to the surface of MNPs (Figure 2D) or forming mCLEA-CALB (Figure 2E) resulted in MNPs showing more fuzzy and diffuse edges than the naked counterparts.

**Figure 3:**
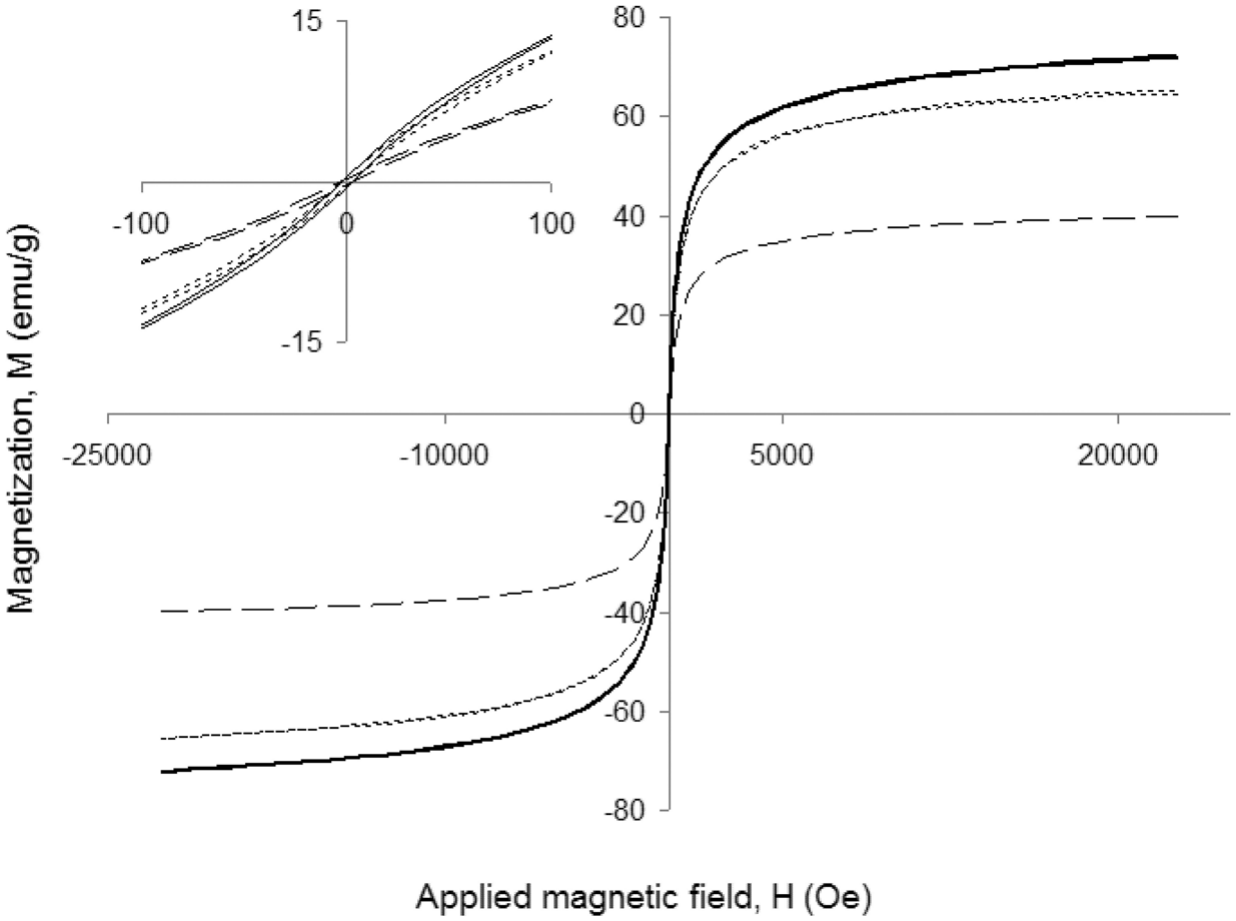
**Magnetization analysis at 300 K of the magnetic biocatalysts obtained with CALB**. MNP-CALB (solid lines), mCLEA-CALB with 25 μ g protein/mg MNPs (dotted lines with short strokes) and mCLEA-CALB with 100 μ g protein/mg MNPs (dotted lines with long strokes). Inlet shows a detail of magnetization for values of magnetic field near to 0 Oe.

**Figure 4:**
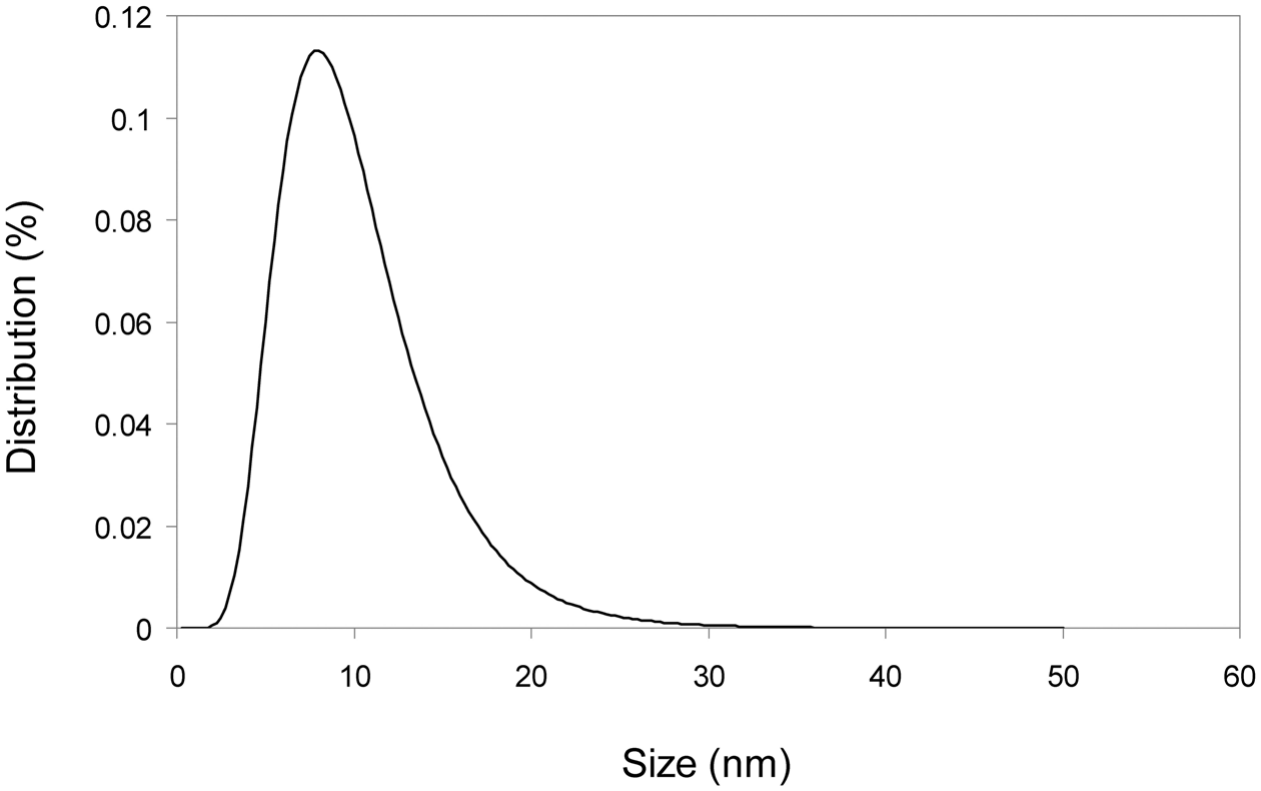
**Particle size distribution of MNPs**. The distribution was fitted to the Langevin's function yielding a mean particle size of 9.5 nm and dispersion of ±6 nm.

Trying to determine if the binding of the protein could modify the magnetic properties of the particles, magnetic behavior of both protein-free MNP-NH_2_ and immobilized enzymes (MNP-CALB and mCLEA-CALB) was analyzed at different magnetic fields and at 300 K (see Figure 3). At this temperature, the shape of the magnetization curve vs. magnetic field was similar for the three biocatalysts. In the three cases, when the external magnetic field was removed the particles lost the magnetization. Also, once saturation reached, magnetization only disappeared when magnetic field was reduced to zero. These two properties are represented by the concepts of magnetic remanence (*M*_*r*_) and coercivity (*H*_*c*_), which were very close to zero, being a characteristic of superparamagnetic nanoparticles. The saturation magnetization (*M*_*s*_) of MNP-NH_2_ was 82.5 emu/g (Table 1), similar to the value corresponding to bulk magnetite (89 emu/g, Ramírez and Landfester, 2003). The presence of cross-linked enzyme lowered the value of *M*_*s*_, and that reduction increased at higher protein concentration, because the saturation magnetization value was referred to the total mass of biocatalyst instead of the mass of the magnetic support. Fitting magnetic results to the Langevin's function, particle size distribution of naked MNPs was obtained. Figure 4 shows a mean nanoparticle diameter of 9.5 nm with a dispersion of ±6 nm.

**Table 1:** **Magnetic parameters of MNP-NH_2_ and biocatalysts obtained of CALB**.

**Support or biocatalyst**	**Immobilized CALB (μg/mg MNPs)**	***M*_***s***_ (emu/g)**	***M*_***r***_ (emu/g)**
MNP-NH_2_	–	82.5	1.0
MNP-CALB	25	65.3	0.5
mCLEA-CALB	25	72.1	0.5
	100	40.0	0.3

### Catalytic properties of free and immobilized CALB

The use of magnetic supports for immobilization of enzymes provides well-known facilities for the recovery and reuse of the catalysts. The benefits in terms of catalytic properties were checked using CALB as model enzyme and three types of immobilization: MNP-CALB (50 μ g protein/mg MNPs), mCLEA-CALB with low load of protein (25 μ g protein/mg MNPs), and mCLEA-CALB with high load of protein (100 μ g protein/mg MNPs) (Table 2). Using non-aggregated enzyme, 50% of the protein was measured in the liquid phase, indicating that only 25 μ g protein/mg MNPs was firmly attached to the support. However, using covalent CLEAs the immobilization efficiency reached 100%.

**Table 2:** **Main properties of magnetic biocatalysts obtained of CALB**.

**Property**		**Free lipase**	**MNP-CALB**	**mCLEA-CALB**	
Immobilized CALB (μg/mg MNPs)		–	25	25	100
Activity^a^	Esterase (*U*/mg catalyst)	20	0.38	0.27	1.0
	Hydrolytic (*U*/mg catalyst)	1416.2	0.2	7.2	28.8
	Transesterification (*U*/mg catalyst)	–	2.0	2.6	6.5
	Biodiesel conversion (%)	–	20.5	30.3	66.7
Stability^b^	Biodiesel conversion after 10 cycles (%)	–	9.4	13.7	59.3

a Activities were assayed as detailed in Materials and Methods.

b Stability was assessed as a measure of the biodiesel (FAPEs) conversion after 10 consecutive catalytic cycles of 24 h at 40°C.

The effect of immobilization was assessed by the measurement of three enzymatic activities using preparations of the free and immobilized biocatalysts: (i) esterase activity using *p*NPA which is converted to *p*NP, (ii) hydrolysis of tributyrin to butyric acid, and (iii) the transesterification of olive oil with propanol to obtain FAPEs.

Having in mind the concentration of immobilized CALB (μ g protein/mg MNPs), esterase activities of free and immobilized biocatalysts can be compared in terms of specific activities (*U*/mg protein): 20, 15.2, 10.8, and 10 *U*/mg protein for free enzyme, MNP-CALB and mCLEA-CALB of low and high load, respectively. The decrease in specific activity of immobilized enzymes compared to free enzymes could be associated to diffusional limitations which usually occur in immobilized biocatalysts due to steric hindrance. In that case, the rate of diffusion of substrates and/or products to and from the active site of the enzyme is lower than the enzymatic reaction rate (Pěsić et al., 2012). These diffusional problems were higher when using CLEAs than for non-aggregated enzyme, which indicates that enzyme aggregation could hinder the diffusion of compounds. The difference between free and immobilized enzyme activities was even more evident when analyzing the hydrolysis of tributyrin, due to the very fast enzymatic reaction.

mCLEA-CALB containing 100 μg protein/mg MNPs resulted to be the most appropriate biocatalyst in terms of enzymatic activities (*U*/mg MNPs), including the transesterification of olive oil to biodiesel, where a very high conversion was obtained after 24 h (Table 2). Moreover, the biocatalyst was much more stable than the other immobilized enzymes tested, maintaining practically the same conversion to biodiesel after 10 reaction cycles. Thermal stability was also considerably higher, as the biocatalyst only lost 10% of the initial activity when heated at 60°C for 30 min, while MNP-CALB and mCLEA-CALB (25 μg protein/mg MNPs) lost 70 and 50%, respectively. In any case, the immobilized biocatalysts were much more stable than the free enzyme, which lost 95% of the initial activity when heated at 60°C for 30 min. MNPs attached to previously aggregated and cross-linked enzymes (mCLEAs) represent the best option for the synthesis of magnetic biocatalysts, as high concentrations of protein can be attached to the enzyme, and this is more protected against destabilizing agents.

### Synthesis of biodiesel

**Figure 5:**
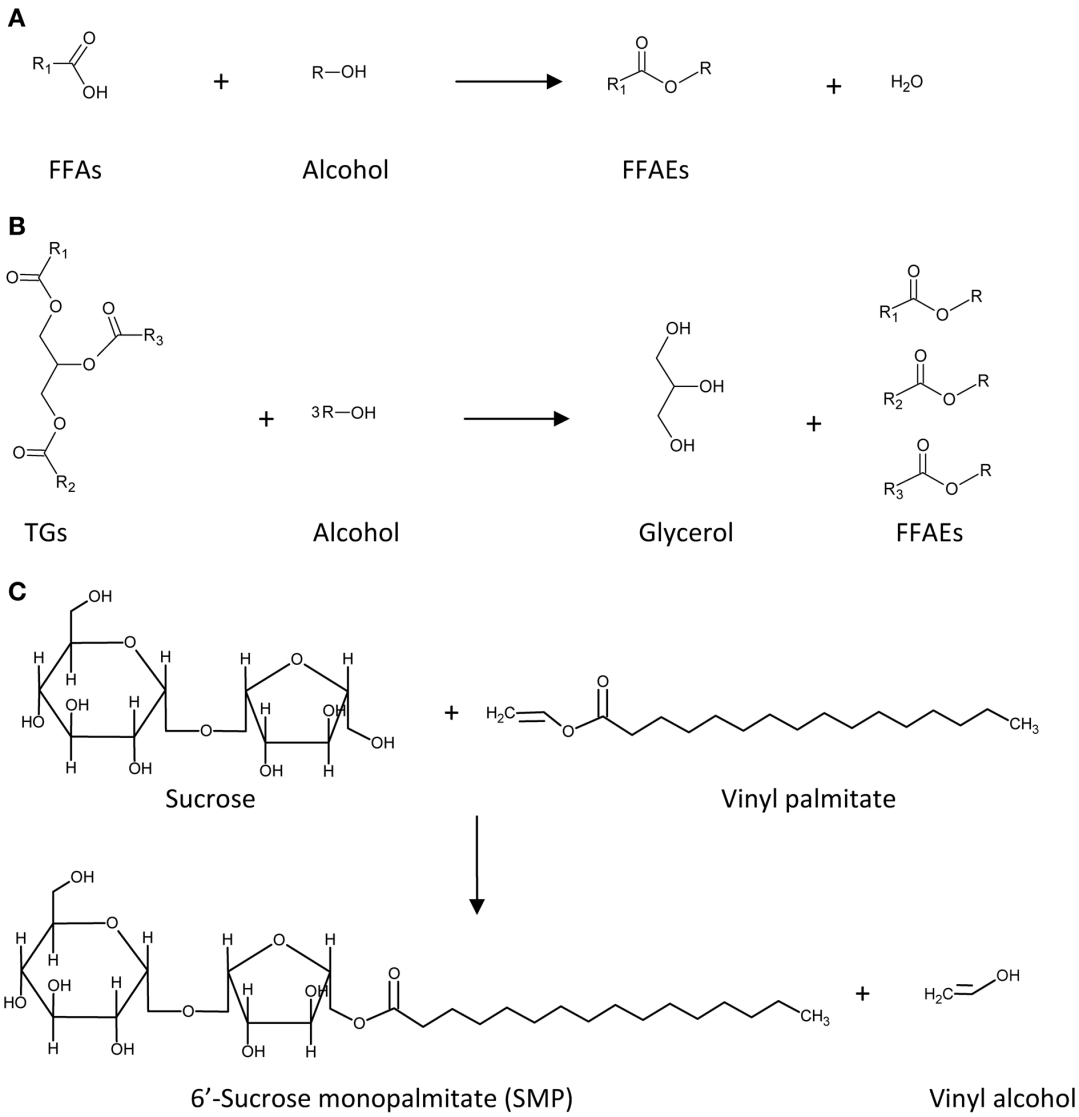
**Reactions of (trans)esterification catalyzed by CALB**. Synthesis of biodiesel from **(A)** free fatty acids (FFAs) and **(B)** triglycerides (TGs). Synthesis of biosurfactant **(C)**.

The ability of the prepared magnetic biocatalysts to synthesize biodiesel was tested using preparations of both FFAs and vegetable oils in a solvent-free system (see Figure 5).

**Figure 6:**
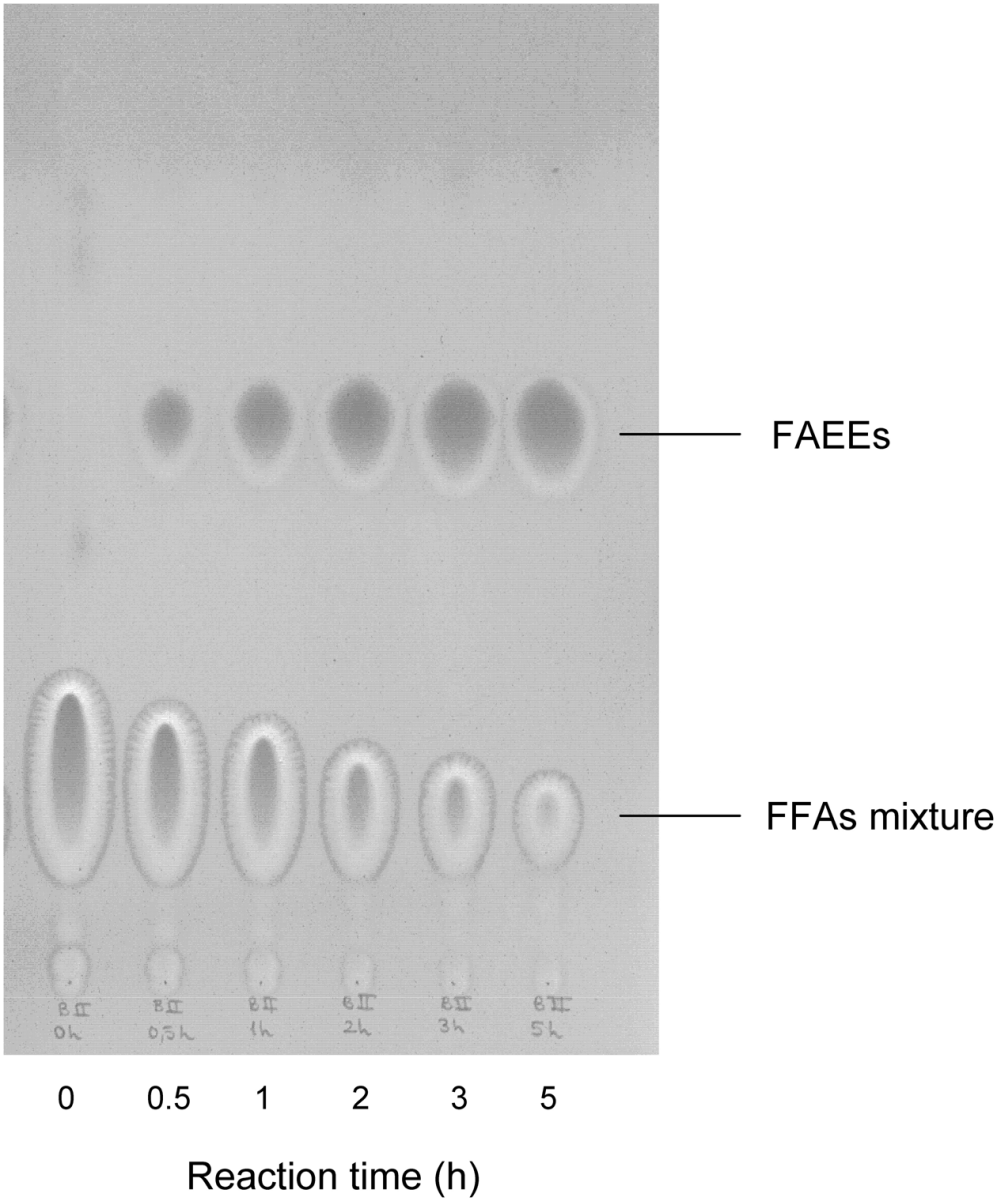
**Analysis by TLC of biodiesel (FAEEs) conversion from a FFA mixture using mCLEA-CALB**. The reaction mixture containing 500 μmol FFA (4.1% stearic acid, 23.6% palmitic acid, 52.1% oleic acid, 12.4% linoleic acid, and 7.8% linolenic acid), ethanol (10:1 alcohol:FFA molar ratio) and 2 mg mCLEA-CALB was incubated for 5 h at 30°C with rotational mixing.

**Figure 7:**
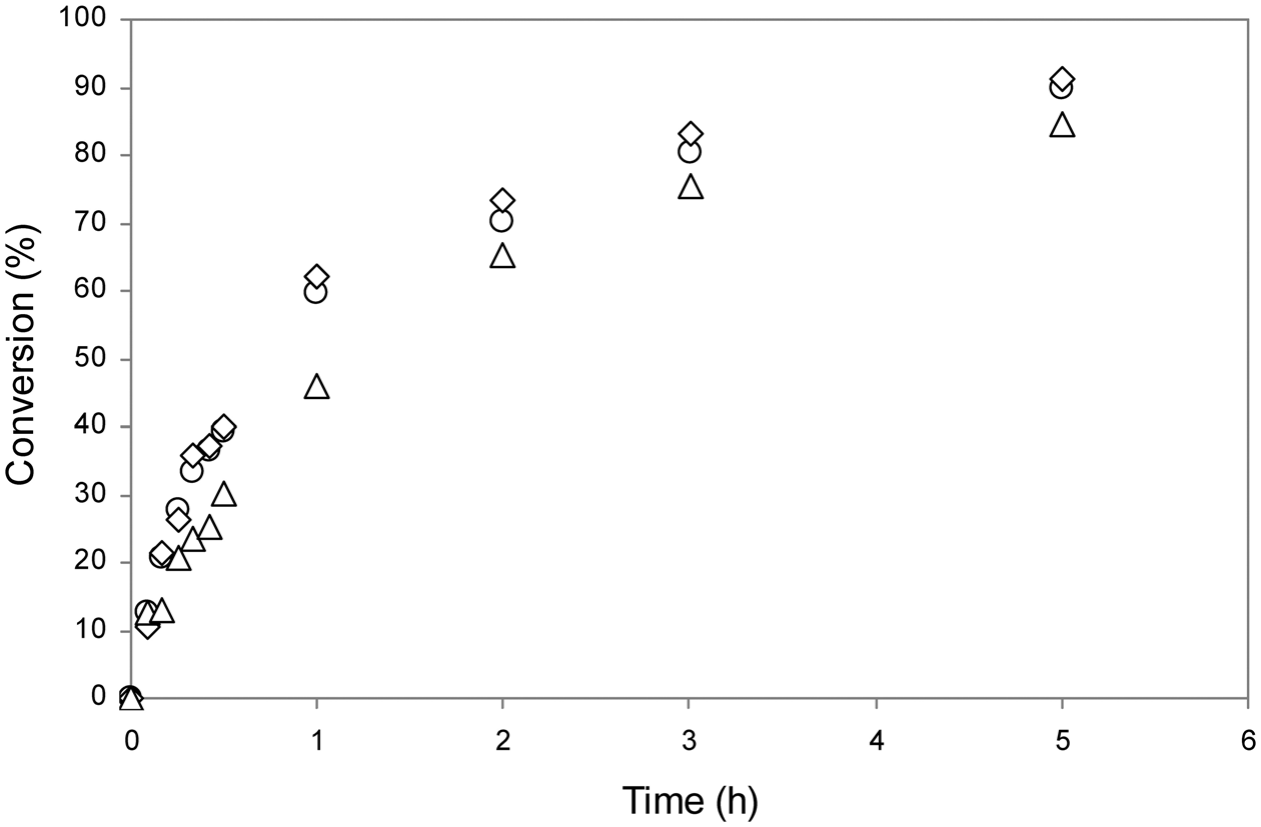
**Analysis by HPLC of biodiesel (FAEEs) conversion from a FFA mixture using mCLEA-CALB**. The reaction conditions were the same as indicated in Figure 6. Conversion of oleic acid (triangles), linoleic acid (diamonds), and linolenic acid (circles).

The esterification of FFAs was performed using a mixture of them which mimics the composition of reserve lipids present in the microalga *Scenedesmus* sp., which mainly consisted of C14 and C16 saturated and unsaturated fatty acids. Ethanol was selected as alkyl donor in a molar ratio of 10:1 (alcohol:FFA), acting also as solvent of FFAs. The reaction (Figure 5A) was followed by both TLC and HPLC, and results are shown in Figures 6, 7, respectively. TLC plates stained with Coomassie Blue is a useful tool to identify and semi-quantify not only FAEEs but also FFAs. Figure 6 reveals that the esterification of FFAs was noticeable at 30 min, and was practically completed after 5 h of reaction. These values were confirmed by HPLC-quantification of FAEEs (data not shown) and unsaturated FFAs (Figure 7), which indicated conversions of 60% for C16:2 and C16:3 at 30 min and 90% after 5 h of reaction.

**Figure 8:**
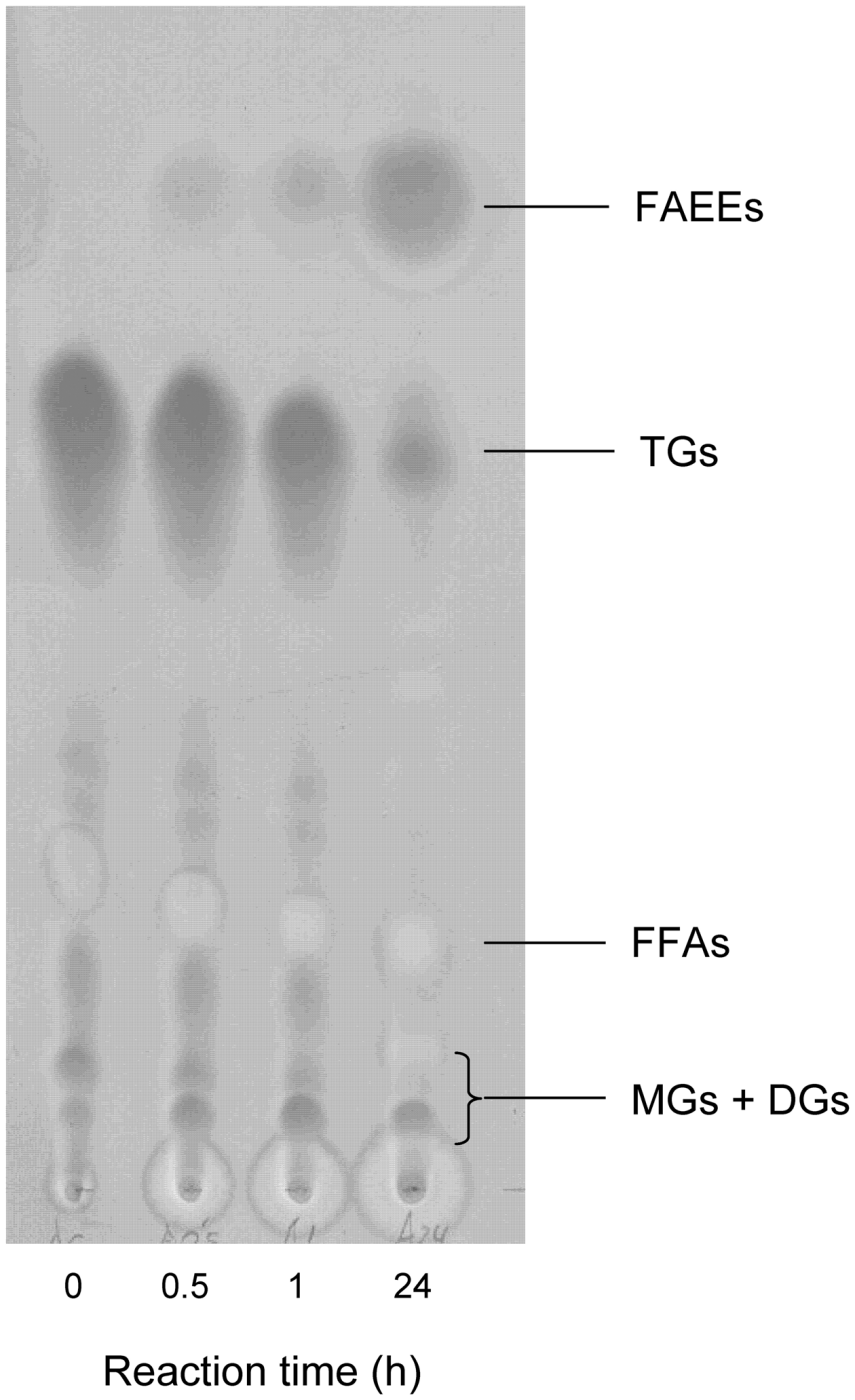
**Analysis by TLC of biodiesel (FAEEs) conversion from vegetable oils mixture using mCLEA-CALB**. The reaction mixture containing 0.2 g oil (80% palm oil, 10% soybean oil, and 10% olive oil), ethanol (30:1 alcohol:oil molar ratio) and 1% (w/w of oil) mCLEAs was incubated for 24 h at 30°C with rotational mixing. Triglycerides (TGs), diglycerides (DGs), monoglycerides (MGs), and free-fatty acids (FFAs).

Trying to improve the simulation of microalgae oil, a mixture of vegetable oils (80% palm oil, 10% soybean oil and 10% olive oil) was prepared and used as substrate for the transesterification reaction. Taking into account the stoichiometry of the reaction (3:1, Figure 5B) and the molar excess of ethanol considered for FFAs, a molar ratio of 30:1 (alcohol:oil) was selected for the transesterification of this oil mixture. TLC analysis of the initial sample (Figure 8, lane 1) indicates the presence of triglycerides, but also the perceptible presence of FFAs, monoglycerides and diglycerides. After 1 h, the spot corresponding to triglycerides decreased, and after 24 h a high amount of triglycerides was converted to FAEEs. The presence of FFAs not only did not interfere in the transesterification of the oil but also they were converted to FAEEs in the reaction catalyzed by CALB. This point is remarkable because, as it is well-known, the presence of FFAs drastically reduces the yield and quality of the product obtained by some of the usual chemical processes applied for the synthesis of biodiesel (Leung et al., 2010). On the other hand, the diversity and complexity of the oil mixture made the transesterification rate significantly lower than the rate of FFAs esterification (Figure 6).

**Figure 9:**
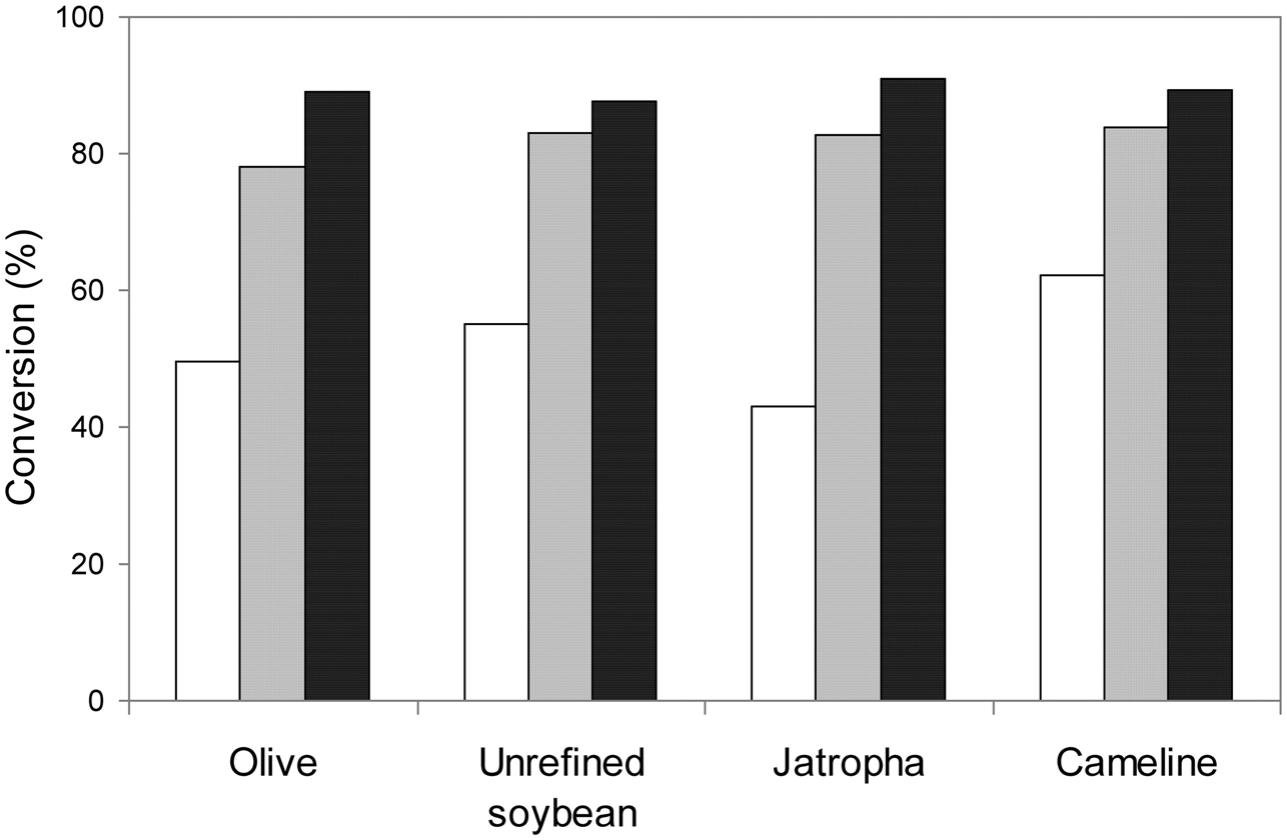
**Analysis by TLC of biodiesel (FAEEs) conversion from different non-edible vegetable oils catalyzed by magnetic biocatalysts of CALB**. The reaction mixture containing 0.2 g oil, 2-propanol (6:1 alcohol:oil molar ratio) and 1% (w/w of oil) of catalyst was incubated for 72 h at 30°C with rotational mixing. MNP-CALB (white bars), mCLEA-CALB with 25 μ g protein/mg MNP (gray bars), and mCLEA-CALB with 100 μ g protein/mg MNP (black bars). Olive oil was used as a control.

Finally, the benefits of mCLEA-CALB as biocatalyst for the transesterification of vegetable oils were pointed out by comparing the three synthesized magnetic CALB catalysts for the synthesis of biodiesel from olive (used as a control), unrefined soybean, jatropha and cameline oils (Figure 9). The behavior of the biocatalysts seemed to be independent of the non-edible oil selected. The comparison of MNP-CALB with mCLEA-CALB of the same protein concentration revealed that the formation of CLEAs before immobilization contributes to the stabilization of the enzyme, and consequently, its maximal exploitation during the enzymatic reaction. Even so, the best conversion results were obtained when the highest concentration of protein was immobilized, reaching biodiesel conversion over 90% in 24 h at 30°C.

### Synthesis of biosurfactants

The enzymatic synthesis of SFAEs is usually limited by its low productivity, as the reaction only occurs in a medium where polar sugar and non-polar fatty acid donor could be soluble. Some attempts were performed to find an adequate medium, which must fulfill solubilization requirements and preserve biocatalyst activity. Commercial immobilized CALB (Novozyme 435) was applied for this purpose with low productivities (Ferrer et al., 1999).

**Figure 10:**
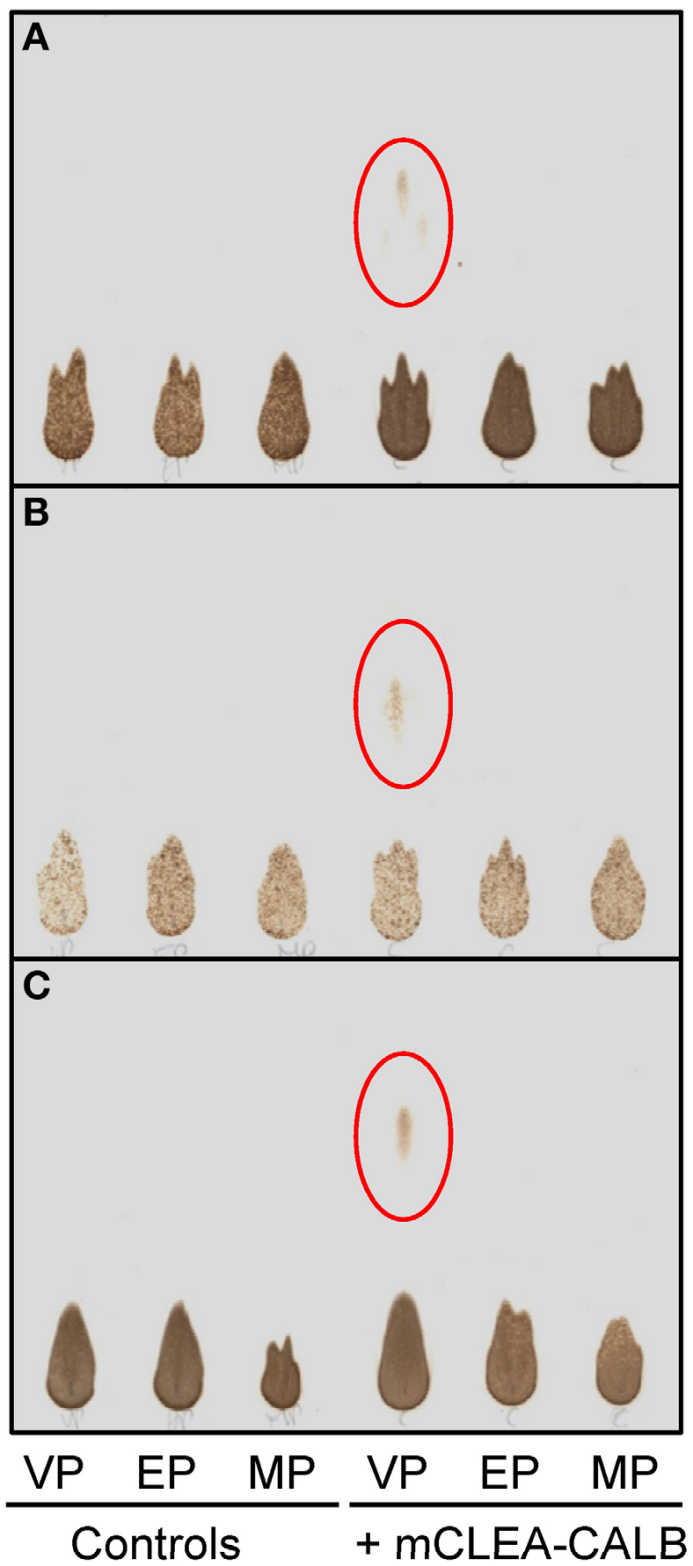
**Analysis by TLC of the SMP conversion from sucrose and different alkyl palmitates catalyzed mCLEA-CALB**. The reaction mixture containing 100 mg/ml sucrose (292 mM), alkyl palmitate and 4 mg/ml mCLEA-CALB in 1 ml DMSO was incubated for 24 h at 60°C. Vinyl palmitate (VP), methyl palmitate (MP), and ethyl palmitate (EP) were used. Sucrose:alkyl palmitate molar rates were 1:1 in **(A)**, 1:2 in **(B)**, and 1:3 in **(C)**.

Taking advantage of the high stability showed by mCLEA-CALB for the synthesis of biodiesel, the utility of this magnetic separable biocatalyst was tested for the transesterification of sucrose with different alkyl palmitate esters (vinyl, methyl and ethyl palmitate) as shown in Figure 5C. DMSO was selected as solvent due to its polar aprotic characteristic, dissolving both polar and non-polar compounds. The palmitate ester was added to 292 mM sucrose dissolved in DMSO in molar ratios sucrose:palmitate of 1:1, 1:2, and 1:3, and the enzymatic reaction using mCLEA-CALB was performed at 60°C for 24 h. Figure 10 shows the results obtained by TLC analysis of the samples. After staining the plates with urea solution, the spots corresponding to sucrose and SMP became visible. Spots corresponding to SMP appeared only when vinyl palmitate was used as fatty acid donor, and the highest concentration corresponded to a molar ratio of 1:3. This result is consistent with previous studies, which point out that the vinyl alcohol formed during the process could tautomerize to the low-boiling-point acetaldehyde, shifting the equilibrium toward the ester formation. However, by employing alkyl fatty acid esters the transesterification reactions became reversible, resulting in low yields (Cruces et al., 2001).

Studies of soluble CALB and mCLEA-CALB stabilities in different polar and non-polar solvents revealed that the magnetic biocatalyst is much more stable than the free enzyme, although the stability in DMSO is still low (the half life of the magnetic enzyme is below 3 h, data not shown). Other solvents were tried in order to enhance enzyme stability, and the solubility losses were overcome with intense magnetic agitation. Results of reaction performed in 2M2P were compared to those obtained using DMSO in Table 3. The presence of DMSO inactivated the enzyme, resulting in a low conversion, which occurred in the first 5 h of reaction. However, taking advantage of stability improvement, mCLEA-CALB catalyzed the production of 40 mM SMP (23 g/l) using 2M2P as solvent.

**Table 3:** **Effect of the solvent used on the production of sucrose 6′-monopalmitate (SMP)^a^**.

**Time (h)**	**SMP (mM)**	
	**Dimethylsulfoxide**	**2-Methyl-2-propanol**
0	0	0
5	0.39	1.59
72	0.40	24.1
168	0.40	39.6

a The reaction mixture (5 ml) contained sucrose (292 mM) and vinyl palmitate (876 mM) in the presence of the solvents DMSO or 2M2P. mCLEA-CALB (20 mg) was used to catalyze the reaction at 60°C with magnetic stirring. At the indicated times samples were withdrawn and analyzed by HPLC-MS.

## Conclusions

In this paper we report that robust magnetically-separable biocatalysts of lipase show higher stability and better performance than the soluble enzymes. Moreover, they can be reused after easily recovery by a magnetic field avoiding the use of filtration or centrifugation which inevitably led to enzyme clumping. We have demonstrated the utility of these biocatalysts to catalyze reactions in both aqueous and non-aqueous media to obtain bioproducts of interest such as biodiesel or biosurfactants from sustainable and renewable sources. These results indicate that synergism of using properly functionalized magnetic nanosupports combined with suitable selection of the adequate enzyme can lead to the development of novel robust magnetic nanobiocatalyts of interest for industry. Enzyme inactivation by solvents or byproducts during bioproducts obtainment can be minimized or avoided by searching for a robust enzyme. The combination of nanotechnology and biocatalysis represents a promising opportunity to develop novel stable and efficient magnetic biocatalysts which can be easily recovered from the reaction mixture and reused in new catalytic cycles thus greatly improving the economic viability for its use to obtain bioproducts at industrial scale.

### Conflict of interest statement

The authors declare that the research was conducted in the absence of any commercial or financial relationships that could be construed as a potential conflict of interest.
